# Demographic, clinical, and socioeconomic factors associated with delayed diagnosis and management of pediatric testicular torsion in West China: a retrospective study of 301 cases in a single tertiary children’s hospital

**DOI:** 10.1186/s12887-021-03001-7

**Published:** 2021-12-06

**Authors:** Chengjun Yu, Jie Zhao, Jiandong Lu, Yi Wei, Li Jiang, Tianxin Zhao, Tao Lin, Dawei He, Sheng Wen, Shengde Wu, Guanghui Wei

**Affiliations:** 1 Department of Urology, Children’s Hospital of Chongqing Medical University, Zhongshan 2nd Road, Yuzhong District, Chongqing, 400014 China; 2 Chongqing Key Laboratory of Children Urogenital Development and Tissue Engineering, Chongqing, China; 3 National Clinical Research Center for Child Health and Disorders, Chongqing, China; 4 China International Science and Technology Cooperation Basfe of Child Development and Critical Disorders, Chongqing, China; 5 Chongqing Key Laboratory of Pediatrics Chongqing, Chongqing, China; 6 Department of Urology, The First Affiliated Hospital of Chongqing Medical University, Chongqing, China; 7 Department of Pediatric Urology, Guangzhou Women and Children’s Medical Center, Guangzhou Medical University, Guangzhou, Guangdong China; 8 Ministry of Education Key Laboratory of Child Development and Disorders, Room 806, Kejiao Building (NO.6), No.136, Zhongshan 2nd Road, 400014, Yuzhong District, Chongqing City, China

**Keywords:** Delayed surgery, Pediatrics, Socioeconomic factors, Testicular torsion

## Abstract

**Background:**

To investigate the association between geographic, clinical, socioeconomic factors and delayed management of pediatric testicular torsion (TT) in West China.

**Methods:**

A retrospective study was conducted on TT at Children’s Hospital of Chongqing Medical University in West China from November 2004 to December 2020. Univariate analysis and logistic regression analysis were conducted to determine the association between these factors and delayed management of TT.

**Results:**

A total of 301 cases were included in this study. The misdiagnosis rate of TT in primary, secondary healthcare units and tertiary hospitals was 93.8, 71.1, and 8.9%, respectively. Approximately 26.9% of TT boys received timely surgical management (within 12 h from symptoms inset to surgery). Logistic regression analyses suggested the following factors were associated with delayed repair of TT:

age less than 6 years (*P* = 0.001), with a history of symptoms progress (*P* = 0.001) or former treatment (*P* <0.001), absence of other diagnosis (*P* = 0.011) and those boys living far away from the main city zones (*P* <0.001).

**Conclusions:**

Delayed surgical management for TT was more likely for boys with age less than 6 years, the absence of other diagnosis, with a history of former treatment or symptoms progress, and those living far away from the main city zone. To maximize the possibility of timely surgical management for TT, it is vital to strengthen the public awareness of TT and conduct continuously re-education and update physicians working at primary and secondary healthcare units.

## Background

Testicular torsion (TT) is an urological emergency and needs prompt management. The incidence rate of TT in pediatrics is about 3.8–5.9 per 100,000, TT is not common but a time dependent urgent event [1–3]. Delay in diagnosis and management of TT is associated with a poor clinical outcome, which may lead to testicular atrophy or even totally loss of the affected testis [1]. To data, there is no controversy that the time between onset of symptoms and detorsion, and the degree of cord twisting are the two main determinants for testicular salvage [4, 5]. An early study from Greece reported that no atrophied testis was observed in TT patients with incomplete torsion (180°–360°) and symptom duration less than 12 h, while absent or severely atrophied testis was observed in all TT cases with complete torsion (> 360°) and duration more than 24 h [6].

A large number of studies reported that, worldwide, the delayed diagnosis and management of TT was quite common, and a relatively high proportion of TT patients proven to be delayed even in tertiary hospitals [7, 8]. Aaron P. Bayne et al. [1] and Christopher E. Bayne et al. [9], in the United States, and Ekici et al. [10] in Turkey, have investigated the clinical, socioeconomic and other factors associated with delayed management of TT, while there have no relevant reports among Chinese populations. Identifying the factors that cause delayed management of pediatric TT in China would help to establish targeted medical health-care plans, salvage affected testes, and eventually protect boys’ reproductive health.

The aim of this retrospective study is therefore to investigate the geographic, clinical, and socioeconomic factors associated with delayed management of pediatric TT in West China. This study consecutively investigated all the surgically confirmed TT cases regarding clinical and socioeconomic factors and delayed surgical repair at the largest children’s medical center in West China from November 2004 to December 2020, and gave further evidence on the correlation between the surgical findings and the duration of symptoms onset to surgery.

## Methods

### Patients demographics, factors, and definitions

A retrospective review of the medical records on TT at the Children’s Hospital of Chongqing Medical University from November 2004 to December 2020 was conducted. Only surgically confirmed TTs and boys age more than six months were consecutively included in this study, these patients refused to surgery or surgically found not to be TT were not included.

According to European Association of Urology guidelines on pediatric urology 2020 (available at https://uroweb.org/guideline/paediatric-urology), surgical detorsion of TT not exceed 12 h was found to avoid infertility, and all TT cases should undergo urgent surgical exploration within 24 h from symptoms onset to diagnosis. We defined delayed management of TT as duration more than 12 h from symptoms onset to surgery, in the meanwhile, we also identified 24 h as an important duration interval.

Demographic data included data of birth, data of symptoms onset, and data of surgery. Age at symptoms onset, and duration of symptoms onset to surgery were calculated by the data of birth, and the time of symptoms onset and surgery. Moreover, age category was further divided into three groups: pre-school (0–5 years), school-age (6–12 years), and puberty (13–18 years).

The extracted clinical data included the following: laterality, other diagnoses, whether having concomitant extra-scrotal symptoms, whether having symptoms progress, history of former treatment, and class of first-consultant medical establishment and diagnosis. Other concomitant diagnoses included hydrocele, cryptorchidism, inguinal hernia, and testicular microlithiasis. Concomitant extra-scrotal symptoms included abdominal pain, nausea, vomit, inguinal pain or masses, and fever. A history of former treatment defined as these patients had a first visit to a primary health care unit or their local hospital before transferring to our hospital. All clinical data were extracted or transferred from reliable medical records.

Socioeconomic information included the following: medical insurance, whether living in poverty counties, whether living in neighboring main city zones around this largest children’s medical center, main guardians, and education degree of guardians. Poverty counties were defined based on the average annual income of the local population, as listed at http://www.cpad.gov.cn/art/2012/3/19/art_50_23706.html. The nine districts in Chongqing City were considered neighboring main city zone. Whether these patients had a medical insurance, who was their main guardians, and the education degree of guardians were asked when these patients checked in. All these messages were recorded from reliable medical records.

In order to figure out the relationship between duration of symptoms onset to surgery and surgical findings of torsed testes, and to, further verify the duration would indicate poor clinical outcomes, we also summarized and compared the differences of surgical findings in different duration intervals. The noticed surgical findings included: direction of rotation, degree of testicular twist, type of torsion, blood supply, grade according to Arda, and outcome of detorsion and sufficient supportive treatments. Grade of Arda was defined as: grade I, sufficient bleeding, bleeding or oozing immediately; grade II, insufficient bleeding, no bleeding immediately after the incision but starting within 10 min; grade III, no bleeding within 10 min [11].

### Surgical management

First, after general anesthesia, the skin and tunica vaginalis of affected side were incised and the testis was reset without tension. Second, the affected testis was detorsed, 1% lignocaine was infiltrated within the spermatic cord. Third, a small incision was made in the tunica albuginea to check the blood supply, and in the meanwhile, a piece of moistened gauze with warm saline was used to cover the testicular tissue for 10–30 min. Forth, the torsed testis was re-examined for potential salvageability. A communication with the guardians during surgery was made to decide whether perform the orchidopexy or orchiectomy of affected testis and fixation of contralateral testis, if coagulative necrosis of the testicular parenchyma was observed. The abovementioned surgical findings were all noted in operation records.

### Statistical methods

All the statistical analyses were performed using the IBM SPSS Statistics for windows, version 25.0 (IBM Corp., Armonk, N.Y., USA). Categorical variables were presented as ratios (%) and continuous variables were presented by mean ± standard deviation (SD) and median (interquartile range). Comparisons were conducted by χ2 test or Fisher’s exact test for dichotomous outcomes and unordered multiple outcomes, by Kruskal-Wallis H test for ordered multiple outcomes. Variables that determined to be significant in the univariate analysis of timely and delayed repair of TT (≤12 h vs >12 h) were included in the logistic regression analysis. A *p*-value less than 0.05 was considered statistically significant.

## Results

Among the potential candidates in this 16-year period, 301 surgically confirmed TT children were included in this study. The number of TT children referred to our center greatly increased in recent 10 years, while the percentage of TT patients received timely management was not improved obviously.

Only about 26.9% of TT boys received timely surgical management (within 12 h). From the univariate analysis of geographic, clinical, and socioeconomic factors associated with delayed surgical management of TT, laterality (*P* = 0.093), medical insurance (*P* = 0.318), identification (*P* = 0.692) and education degree of main guardians (*P* = 0.146) were not different significantly among those TT children who received delayed or timely repair (Table 1). Among the TT boys with delayed surgical management, the percentages of non-comorbidity (*P* = 0.020), with symptoms progress (*P* <0.001), a history of former treatment (*P* <0.001), a first-treat in a primary or secondary health-care unit (*P* <0.001), living in a poverty county (*P* = 0.002), and far away from the main city zones (*P* <0.001) were significantly higher than those percentages among the children who received timely surgical repair of TT. In addition, age was significantly different among TT children with delayed or timely management (*P* = 0.030), and our data indicated that the older the age was, the lower risk of delayed management the boys suffered (Table 1).

**Table 1:** Demographic, clinical, and socioeconomic characteristics of 301 pediatric testicular torsion cases in different duration periods from symptoms onset to surgery

	Total (*n* = 301)	0–12 h (*n* = 81)	13–24 h (*n* = 33)	>24 h (*n* = 187)	*P*	*P*_(≤12h vs >12h)_	*P*_(≤24h vs >24h)_
**Demographic features**							
Age at symptoms onset (month), median (IQR)	144 (76.5163.0)	148 (126.0,164.5)	136 (26.0,162.0)	141 (53,162)	0.020*	0.010*	0.176
Average age (month), mean ± SE	119.9 ± 3.3	137.7 ± 4.98	102.8 ± 11.2	115.3 ± 4.4	0.020*	0.010*	0.176
Age category, n (%)					0.014*	0.030*	0.228
Pre-school (0–5)	71 (23.6)	9 (11.1)	12 (36.4)	50 (26.7)	Ref	Ref	Ref
School-age (6–12)	80 (26.6)	24 (29.6)	9 (27.3)	47 (25.1)	0.034*	0.010*	0.137
Puberty (13–18)	150 (49.8)	48 (59.3)	12 (36.4)	90 (48.1)	0.004*	0.002*	0.134
**Clinical features**							
Laterality, n (%)					0.114	0.093	0.598
Left	232 (77.1)	57 (70.4)	29 (87.9)	146 (78.1)			
Right	69 (22.9)	24 (29.6)	4 (12.1)	41 (21.9)			
Other diagnosis, n (%)					0.003*	0.020*	0.001*
None	110 (36.5)	21 (25.9)	7 (21.2)	82 (43.9)	Ref	Ref	Ref
Cryptorchidism	19 (6.3)	5 (6.2)	1 (10.6)	13 (7.0)	0.41	0.581	0.576
Hydrocele	154 (51.2)	51 (63.0)	23 (69.7)	80 (42.8)	0.001*	0.012*	<0.001*
Ipsilateral	140 (90.9)	49 (96.1)	21 (91.3)	70 (87.5)	<0.001*	0.005*	<0.001*
Contralateral	7 (4.5)	0 (0)	1 (4.3)	6 (7.5)			
Bilateral	7 (4.5)	2 (3.9)	1 (4.3)	4 (5.0)			
Inguinal hernia	3 (1.0)	0 (0)	0 (0)	3 (1.6)			
Testicular microliathiasis	8 (2.7)	2 (2.5)	1 (3.0)	5 (2.7)	0.541	0.486	0.740
Concomitant extra-scrotal symptoms, n (%)					0.181	0.065	0.135
None	243 (80.7)	71 (87.7)	26 (78.8)	146 (78.1)	Ref	Ref	Ref
Abdominal pain	16 (5.3)	1 (1.2)	2 (6.1)	13 (7.0)	0.077	0.089	0.092
Nausea and/or vomit	27 (9.0)	5 (6.2)	4 (12.1)	18 (9.6)	0.465	0.241	0.506
Fever	5 (1.7)	0 (0)	1 (3.0)	4 (2.1)			
Inguinal pain/ mass	10 (3.3)	4 (4.9)	0 (0)	6 (3.2)			
With symptoms progress, n (%)					0.001*	<0.001*	0.003*
Yes	55 (18.3)	4 (4.9)	7 (21.2)	44 (23.5)			
No	246 (81.7)	77 (95.1)	26 (78.8)	143 (76.5)			
History of former treatment, n (%)					<0.001*	<0.001*	<0.001*
Yes	91 (30.2)	3 (3.7)	8 (24.2)	80 (42.8)			
No	210 (69.8)	78 (96.3)	25 (75.8)	107 (57.2)			
Class of first-consultant medical establishment and diagnosis, n (%)					<0.001*	<0.001*	<0.001*
Primary health-care unit	32 (10.6)	1 (1.2)	5 (15.2)	26 (13.9)	Ref	Ref	Ref
Highly suspected TT	2 (6.2)	0 (0)	0 (0)	2 (7.7)			
Misdiagnosis	30 (93.8)	1 (100)	5 (100)	24 (92.3)			
Secondary	45 (15.0)	1 (1.2)	3 (9.1)	41 (21.9)	NA	NA	0.118
Highly suspected TT	13 (28.9)	0 (0)	2 (66.7)	11 (26.8)			
Misdiagnosis	32 (71.1)	1 (100)	1 (33.3)	30 (73.2)			
Tertiary	224 (74.4)	79 (97.5)	25 (75.8)	120 (64.2)	0.001*	P<0.001*	0.003*
Highly suspected TT	204 (91.1)	74 (93.7)	22 (88.0)	108 (90.0)			
Misdiagnosis	20 (8.9)	5 (6.3)	3 (12.0)	12 (10.0)			
**Socioeconomic features**							
Medical insurance, n (%)					0.133	0.318	0.061
Yes	183 (60.8)	53 (65.4)	24 (72.7)	106 (56.7)			
No	118 (39.2)	28 (34.6)	9 (27.3)	81 (43.3)			
Poverty county residence, n (%)					0.004*	0.002*	0.001*
Yes	42 (14.0)	3 (3.7)	3 (9.1)	36 (19.3)			
No	259 (86.0)	78 (96.3)	30 (90.9)	151 (80.7)			
Neighboring main city zone, n (%)					<0.001*	<0.001*	<0.001*
Yes	143 (47.5)	62 (76.5)	21 (63.6)	60 (32.1)			
No	158 (52.5)	19 (23.5)	12 (36.4)	127 (67.9)			
Main guardian, n (%)					0.886	0.692	0.969
Parents	277 (92.0)	74 (91.4)	31 (93.9)	172 (92.0)			
Grandparent	13 (4.3)	4 (4.9)	1 (3.0)	8 (4.3)			
Near relation	11 (3.7)	3 (3.7)	1 (3.0)	7 (3.7)			
Degree of guardians’ education, n (%) ^&&^					0.343	0.146	0.257
Primary school	27 (20.3)	5 (12.2)	3 (23.1)	19 (24.1)			
Secondary school	53 (39.8)	14 (34.1)	6 (46.2)	33 (41.8)			
Senior high school	25 (18.8)	11 (26.8)	3 (23.1)	11 (13.9)			
College and above	28 (21.1)	11 (26.8)	1 (7.7)	16 (20.3)			

*IQR* indicates interquartile range, *SE* standard error of the mean, *TT* testicular torsion

*P ≤ 0.05

^&&^ Only 133 guardians’ degree of education available since 2014

All of the variables that determined to be statistically significant by the univariate analysis between TT children who received delayed or timely management were included in the logistic regression analysis (Table 2). Boys with age less than 6 years (*P* = 0.001), with a history of symptoms progress (*P* = 0.001) or former treatment (*P* <0.001) were significantly more likely to receive a delayed surgical management than a timely one. Patients without other diagnosis (*P* = 0.011) and those boys living far away from the main city zones (*P* <0.001) also associated with a delayed repair.

**Table 2:** Logistic regression analysis of factors associated with delayed surgical management for pediatric testicular torsion (more than 12 h from symptoms onset to surgery)

Factors	Parameter estimate	SE	*P*	OR (95% CI)
Age less than 6 years	1.493	0.433	0.001	4.458 (1.906–10.425)
Without other diagnosis	0.873	0.342	0.011	2.393 (1.225–4.676)
Away from the main city zone	1.713	0.340	<0.001	5.543 (2.844–10.804)
History of former treatment	2.421	0.561	<0.001	11.262 (3.754–33.788)
With symptoms progress	1.972	0.581	0.001	7.186 (2.302–22.435)

*SE* standard error of the parameter estimate, *OR* odds ratio, *CI* confidence interval

In surgical findings, the direction of rotation and type of torsion were not significantly different between those boys who received a delayed repair or not (Table 3). However, the degree of cord twisting, the blood supply, and grade of Arda, and outcomes of detorsion were significantly much different among boys received delayed management or timely repair (*P* <0.001).

**Table 3:** The association between duration periods from symptoms onset to surgery and intraoperative findings

	Total (n = 301)	0–12 h (n = 81)	13–24 h (n = 33)	>24 h (n = 187)	*P*	*P*_(≤12h vs >12h)_	*P*_(≤24h vs >24h)_
Direction of rotation, n (%)					0.816	0.543	0.552
Clockwise	181 (60.1)	51 (63.0)	20 (60.6)	110 (58.8)			
Anticlockwise	120 (39.9)	30 (37.0)	13 (39.4)	77 (41.2)			
Degree of cord twisting, n (%) °					0.145	0.050*	0.123
0–180	44 (14.6)	22 (27.2)	5 (15.2)	17 (9.1)	Ref	Ref	Ref
181–360	104 (34.6)	23 (28.4)	9 (27.3)	72 (38.5)	0.002*	0.001*	0.001*
361–540	67 (22.3)	14 (17.3)	10 (30.3)	43 (23.0)	0.014*	0.005*	0.007*
541–720	80 (26.6)	20 (24.7)	9 (27.3)	51 (27.3)	0.006*	0.001*	0.008*
>720	6 (2.0)	2 (2.5)	0 (0)	4 (2.1)			
Type of torsion, n (%)					0.787	0.625	0.491
Extra-vaginal	21 (7.0)	7 (8.6)	2 (6.1)	12 (6.4)			
Intra-vaginal	280 (93.0)	74 (91.4)	31 (93.9)	175 (93.6)			
Blood supply, n (%)					<0.001*	<0.001*	<0.001*
Rich	73 (24.2)	49 (60.5)	12 (36.4)	12 (6.4)	Ref	Ref	Ref
Poor	84 (27.9)	24 (29.6)	9 (27.3)	51 (27.3)	<0.001*	<0.001*	<0.001*
Little	144 (47.8)	8 (9.9)	12 (36.4)	124 (66.3)	<0.001*	<0.001*	<0.001*
Grade of Arda, n (%) ^ΓΓ^					<0.001*	<0.001*	<0.001*
I	27 (9.0)	20 (24.7)	4 (12.1)	3 (1.6)	Ref	Ref	Ref
II	120 (39.9)	51 (63.0)	14 (42.4)	55 (29.4)	0.003*	0.003*	0.001*
III	154 (51.2)	10 (12.3)	15 (45.5)	129 (69.0)	<0.001*	<0.001*	<0.001*
Outcome of detorsion and adequate management, n (%)					<0.001*	<0.001*	<0.001*
Obviously improved	67 (22.3)	45 (55.6)	8 (24.2)	14 (7.5)	Ref	Ref	Ref
Slightly improved	85 (28.2)	27 (33.3)	10 (30.3)	48 (25.7)	<0.001*	<0.001*	<0.001*
Not improved	39 (13.0)	7 (8.6)	7 (21.2)	25 (13.4)	<0.001*	<0.001*	<0.001*
Orchiectomy	110 (36.5)	2 (2.5)	8 (24.2)	100 (53.5)	<0.001*	<0.001*	<0.001*

^ΓΓ^_Grade I, sufficient bleeding, i.e. bleeding or oozing when the biopsy was obtained; Grade II, insufficient bleeding, no bleeding immediately after the incision but starting within 10 min; Grade III, no bleeding within 10 min_

*P ≤ 0.05

## Discussion

Our study investigated the possible geographic, clinical, and socioeconomic factors that may cause delayed surgical management of pediatric TT, which was rarely reported in Chinese population. This study concerned the correlation between age, identification and education degree level of guardians, former treatment, absence of extra-scrotal symptoms, and whether a progress of symptoms and the time of duration from symptoms onset to surgery, which was also rarely reported in literatures. The definition of delayed surgical management of TT was still not well established, 12 h and 24 h were the most two discussed boundary [12, 13], and whether less than 12 h was more accepted in latest EAU guidelines pediatric urology 2020. We concerned both time intervals.

The surgical findings in this study furtherly confirmed the delayed management of TT would lead to less activity, insufficient blood supply, and poor outcome of torsed testes after adequate detorsion and supportive treatment. These conditions were all directly associated with subsequent poor clinical outcomes and ability of testicular salvage, consistent with literature findings [1, 14].

According to our study, the misdiagnosis rate of TT in primary, secondary and tertiary hospital was 93.8, 71.1, and 8.9%, respectively (Table 1). The misdiagnosis rate of TT was high, especially in primary and secondary levels-of-healthcare units. There were more than 30% boys with acute scrotum firstly treated not in a tertiary hospital, which led to a delayed surgical management of TT. And in addition, these boys transferred from primary and secondary healthcare units were much more likely to suffer an orchiectomy. Our findings indicated that it was very important to strengthen the public awareness of TT, and effects must be conducted to continuously re-educate and update physicians working at primary and secondary healthcare units. This finding was consistent with two latest similar studies from India and Brazil [7, 8], which indicated that improvement of accurate diagnosis of TT in primary and secondary healthcare units was greatly needed around the world.

Age less than six years was more likely to have delayed management of TT than those elder boys, this may be because small children wouldn’t judge the severity of scrotal uncomfortableness and express themselves. The same way, TT children without other diagnosis (mainly hydrocele) may suffer less pain, reddness and swelling, and this may further delay the treatment. About 52.5% of children lived far away from the Chongqing main city zone, these boys were apt to have their first consultant in the local healthcare units, while the local hospitals were suffering lack of experience on the identification and diagnosis of TT, thus made the delayed surgical management of TT.

Though this study had a relatively large participants in pediatric TT, there still existed several limitations. First, our results were limited by the retrospective observational design nature of the analysis. Some clinical and socioeconomic factors that may be related to delayed surgery, such as family history of TT, family economic conditions, guardians’ work, traffic conditions, were not available. Although our analysis was not full-scale, few studies have concerned these factors in such population and few studies have participants more than 300. Second, all data were drawn from the largest child specialist medical center in West China, some selective bias could not be avoided. In addition, the general public has a preference for treating in tertiary hospitals regardless of the severity of disease [15]. Third, those TT boys who were diagnosed and detorsed not in our hospital were not included into analysis, which may not completely represent the general condition.

## Conclusion

Only approximately 26.9% of TT children received timely surgical management (within 12 h from symptoms onset to surgery) in West China. Delayed surgical management for TT was more likely for boys with age less than 6 years, the absence of other diagnosis, with a history of former treatment or symptoms progress, and those living far away from the main city zone. The misdiagnosis rate of TT in primary and secondary healthcare units was very high. It is vital to strengthen the public awareness of TT and conduct continuously re-education and update physicians working at primary and secondary healthcare units to maximize the possibility of timely surgical management for TT.

## Data Availability

The datasets used and/or analysed during the current study available from the corresponding author on reasonable request.

## Abbreviations

CI: Confidence interval
IQR: Interquartile range
OR: Odds ratio
SE: Standard error
TT: Testicular torsion
